# *Citrus tristeza virus* (CTV) Causing Proteomic and Enzymatic Changes in Sweet Orange Variety “Westin”

**DOI:** 10.1371/journal.pone.0130950

**Published:** 2015-07-24

**Authors:** Milena Santos Dória, Aurizângela Oliveira de Sousa, Cristiane de Jesus Barbosa, Márcio Gilberto Cardoso Costa, Abelmon da Silva Gesteira, Regina Martins Souza, Ana Camila Oliveira Freitas, Carlos Priminho Pirovani

**Affiliations:** 1 Centro of Biotechnologia and Genetica, Universidade Estadual de Santa Cruz, UESC, Rodovia Ilhéus-Itabuna, Km 16, Ilhéus/BA, 45662–000, Brasil; 2 Embrapa Mandioca e Fruticultura, CEP 44380–000, Cruz das Almas, BA, Brazil; Henan Agricultural Univerisity, CHINA

## Abstract

Citrus Tristeza disease, caused by CTV (*Citrus tristeza virus*), committs citrus plantations around the world and specifically attacks phloem tissues of the plant. The virus exists as a mixture of more or less severe variants, which may or may not cause symptoms of Tristeza. The objective of this study was to analyze the changes caused by CTV in the proteome of stems of sweet orange, as well as in the activity and gene expression of antioxidant enzymes. The CTV-infected sweet orange displayed mild symptoms, which were characterized by the presence of sparse stem pitting throughout their stems. The presence of virus was confirmed by RT-PCR. Proteomic analysis by 2DE-PAGE-MS / MS revealed the identity of 40 proteins differentially expressed between CTV- infected and -non-infected samples. Of these, 33 were up-regulated and 7 were down-regulated in CTV-infected samples. Among the proteins identified stands out a specific from the virus, the coat protein. Other proteins identified are involved with oxidative stress and for this their enzymatic activity was measured. The activity of superoxide dismutase (SOD) was higher in CTV-infected samples, as catalase (CAT) showed higher activity in uninfected samples. The activity of guaiacol peroxidase (GPX) did not vary significantly between samples. However, ascorbate peroxidase (APX) was more active in the infected samples. The relative expression of the genes encoding *CAT*, *SOD*, *APX* and *GPX* was analyzed by quantitative real time PCR (RT-qPCR). The CTV-infected samples showed greater accumulation of transcripts, except for the *CAT* gene. This gene showed higher expression in the uninfected samples. Taken together, it can be concluded that the CTV affects the protein profile and activity and gene expression of antioxidant enzymes in plants infected by this virus.

## Introduction

Among the fruits which are consumed raw and used industrially, citrus fruits represent the main world demand [1]. Brazilian economy benefits highly from the farming of those fruits, mainly in respect of the production of oranges, as Brazil stands out as one of its main producers in the world, and is responsible for exporting around US$ 7.8 billion worth of concentrated and frozen orange juice a year. Around 60% of the orange juice consumed in the world comes from Brazilian orchards, which dominate the international market for that fruit [2].

The losses caused by diseases represent a major problem for citriculture. Tristeza is one of those diseases which affect citriculture throughout the world [3]. The disease is caused by *Citrus tristeza virus* (CTV), a member of genus *Closterovirus* which belongs to family *Closteroviridae* [4]. Symptoms caused by CTV may vary, and they depend on the host’s characteristics. Some genotypes are tolerant of the virus, whereas some others are resistant. Resistant plants do not multiply the virus—if they do, it is a much reduced multiplication. In turn, tolerant plants allow for that multiplication, but they tolerate the virus presence in their tissues, but do not develop symptoms of the disease [5].

CTV attacks phloem tissues of plants in *Rutaceae* family, mainly of the genus *Citrus* [4]. In susceptible scion/rootstock combinations, the virus causes classic symptoms, such as mature leaf yellows, rotting of roots, and even plant death. However, in tolerant plants those symptoms do not appear. In that case, stem pitting may be formed. They are flutes or buttresses on the bark [6]. Besides that, the infection causes phloem degeneration in intolerant plants, which may lead them to die [6].

Plants facing biotic stress, such as viral infections, generally react with changes in their protein profile [7] or develop a secondary response, such as increased oxidative stress due to the production of reactive oxygen species (ROS) [8]. When that happens, plants use a set of enzymes and antioxidant substances such as ascorbate, for example, to combat those ROS and to reduce the damage they cause to cells [9]. Virus characteristics [10], existing strains [11] and symptoms of sick plants [4] have already been described, but there are no proteomic studies yet on the Citrus x CTV interaction.

In this study, the protein profiles of non- and infected sweet orange variety “Westin” by CTV varieties were analyzed and compared. Some identified proteins were induced through the interaction between CTV and sweet orange variety “Westin”, and the activity of enzymes involved with the oxidative stress was different when the two treatments were compared. The standard coding gene expression of some of those enzymes was correlated to the enzyme activity, as well as with the standard accumulation of some isoforms identified through ms/ms.

## Materials and Methods

### Plant material and cultivation conditions

Plants were obtained from the Embrapa Mandioca e Fruticultura (EMBRAPA). Samples were obtained from four infected adult plants naturally infected and four non infected adult plants cultivated in a greenhouse with eight years of age in average, of sweet orange variety “Westin” (*Citrus sinensis* (L.). Osbeck.). From each plant, two samples of young stem branches measuring from 0.3 to 0.7 mm in diameter were taken. Those branches were cut in pieces of approximately 5 cm, and immediately inserted in liquid nitrogen. Then, they were freeze-dried and stored at -20°C until the extraction were conducted.

### Evaluation of symptoms

To evaluate the incidence and severity of stem pitting symptoms, 10 branches from each plant used were collected in different parts of plant scions, randomly. They measured approximately 20 cm in length. Those branches were submitted to a temperature of 120°C for 15 minutes in an autoclave. Barks were removed, and branches were then evaluated using a rating scale [12].

### Nucleic acid extraction, synthesis of cDNA, RT-PCR and RT-qPCR

Total RNA was extracted from a pool composed of steam branches from four different plants. The branches were peeled, and barks were macerated in liquid nitrogen and 0.07% / g of Polyvinylpolypyrrolidone (PVPP) tissue. Different rates were used to extract RNA and proteins. RNA extraction was conducted through ZR Plant RNA Miniprep kit (ZymoResearch), according to the manufacturer’s instructions. Sample integrity and quality were confirmed through an analysis with agarose gel at 0.8%. They were qualified in a NanoDrop. Samples were treated with RNAse-Free DNAse (Fermentas Life Science). Two cDNAs were synthesized, one for the semi-quantitative PCR reaction (RT-PCR) and another for the quantitative PCR reaction (RT-qPCR) using Revert Aid H Minus kit (Fermentas—First Strand cDNA Synthesis Kit). For those syntheses, 1 μg RNA in each reaction, a random primer for RT-PCR analysis, and an oligo-dT primer for RT-qPCR analysis were used. Reverse transcription reaction was conducted in a PTC-200 Thermal Cycler (Peltier Thermal Cycler), at 60°C for one hour. After the synthesis, both cDNAs were quantified in a NanoDrop.

The RT-PCR with specific primers for CTV [13] was conducted with cDNA from symptomatic and asymptomatic plants (S1 Table). In order to do that, 3μL of cDNA synthesized for a 50 μL reaction containing 5 units of Taq DNA polimerase, 2.5 mM of MgCl2, 0.8 mM of dNTP, and 5 mM of each sense and antisense primer. The amplification conditions were: 1 step at 94°C for 2 minutes, 30 cycles at 94°C for 30 seconds, 55°C for 30 seconds, and 72° for 45 seconds, followed by a step at 72°C for 5 minutes. PCR products were analyzed through a 1% agarose gel electropheresis.

RT-qPCR was conducted in order to assess the relative abundance of transcripts from some of the genes related to oxidative stress in the plants of each treatment (infected and non-infected branches). RT-qPCR reactions were conducted in five replicates from the tissue pool of four plants, and the volume used in the reaction was 22 μL, containing 100 ng of cDNA, 1 μL of each primer (R+F) 10 μmol.L-1 and 11 μL of Maxima SYBR Green/ROX qRT-PCR Master Mix (2X) (Fermentas). The amplification cycling was conducted in the following steps: 50°C for 2 minutes, 95°C for 10 minutes, 95°C for 15 seconds, and 60°C for 1 minute. These steps were repeated over 40 cycles. After the gene amplification cycling, the products were dissociated in order to confirm the formation of unique and specific products. In this case, the steps conducted presented the following characteristics: 95°C for 15 seconds, 60°C for 1 minute, and 95°C for 1 second. The expression was quantified through the use of method 2^-ΔΔCt^ [14]. The specific primers for each gene are presented in Table 1.

**Table 1:** Sequence of primers used in real-time PCR reactions (qRT-PCR) and in RT-PCR reactions.

Organism	Primer	Sequence (Forward /Reverse)
**Citrus**	**UPL7**	5’CAAAGAAGTGCAGCGAGAGA -3’/5’-TCAGGAACAGCAAAAGCAAG -3’
	**SOD**	5’-TAGGCTTGGGTAAATCCGTAGGA-3’/5’-CTAATCGCCGGCTCCAAAG-3’
	**CAT**	5’-CCGATCACGAGGACCAATTT-3’/5’-TTCAAGACCAAGCGTCCAACT-3’
	**GPX**	5’-GCTTGGAGATTCTGGCCTTTC-3’/5’-TCAGCCTTAAAGCGAGTGCAT-3’
	**APX**	5’-TATTGCCGTTAGGCTTTTGGA-3’/5’-GGTAACCTCAACGCCAACAAC-3’
**CTV**	**CN487/489**	^697^5’- GCGTTGGATGATATCCTTCGCTGG-3’^720^/^1082^5’- AATTRTTCCGCSCAGGACGGAACA-3’^1105^
	**CN488/491**	^1082^5’- TGTTCCGTCCTGSGCGGAAYAATT-3’^1105^/^1461^5’- GTGTARGTCCCRCGCATMGGAACC-’1484

### Protein extraction and quantification

A mass of approximately 0.4 g of the material from the pool of four plants, which were previously and macerated in liquid nitrogen, was used. Proteins were extracted according Pirovani *et al*. [15] using a procedure based on the conduction of successive washings, associated with the sonication steps. Phenol and dense SDS were also used. The precipitated proteins were re-suspended in a rehydration buffer (6 mol.L^-1^ of urea, 2 mol.L^-1^thiourea, 2% CHAPS, and 0.002% of bromophenol blue), and stored at -20°C until used. Proteins were quantified with a 2D-Quant kit (GE HealthCare), as per the manufacturer’s instructions.

### 2—DE and comparative proteome analysis

A mass of 500 μg of proteins was homogenized in a rehydration buffer containing DTT (50 mmol.L^-1^) and ampholytes at 0.5% for pH of 3–10 NL (non-linear) (Amersham Biosciences) with its volume adjusted for 250 μL with the use of a rehydration buffer. The samples were applied on 13-cm gel strips with immobilized pH, which varied from 3 to 10 NL (Amersham Biosciences, Immobiline Dry-Strip). The strips were focused using EttanIPGhor3 (GE Healthcare), which was controlled by software Ettan IPGhor3, as per the following parameters: 12 hours of rehydration at 20°C and approximately 5 hour of focusing (500V- 60 min; 1000V- 64 min; 8000V-150 min; 8000V- 40 minutes). At the end of the focusing process, the strips were placed in a buffer solution (6 mol.L^-1^ of urea, 75 mmol.L^-1^ of Tris-HCl pH 8.8, 30% glycerol, 2% SDS, 0.002% bromophenol blue) a -80°C until they were used.

After the strips were focused, they were treated with DTT (50 mM Tris-HCl buffer (pH 8.8), 6 M urea, 30% glycerol, 2% SDS, 1% dithiothreitol-DTT, a trace of bromophenol blue), and Iodoacetamide (50 mM Tris-HCl buffer (pH 8.8), 6 M urea, 30% glycerol, 2% SDS, 1% iodoacetamide, a trace of bromophenol blue) for fifteen minutes each, and placed on top of a 12% polyacrylamide gel, and sealed with an agarose solution (25 mmol.L^-1^ of Tris base, 192 mmol.L^-1^ of glycine, 0.1% SDS, 0.5% agarose, 0.002% bromophenol blue).

The run was performed until the bromophenol blue arrived at the end of the gel, according the following parameters: (1) 15 mA for 15 minutes, (2) 40 mA for 30 minutes, and (3) 50 mA for 3 hours. After the run, the gels were fixed in a buffer solution containing ethanol 40% and acetic acid 10% for 1 hour, and visualized with 0.08% Coomassie blue G-250 (w/v) [16]. The gels were dyed for 7 days under agitation. After that period, they were discolored through successive washings with distilled water, and then kept in acetic acid at 7%.

### Image acquisition and data analysis

Gel images were obtained through LabScanner (Amersham Bioscience), and analyzed with software Image Master 2D Platinum 7.0 (GE Healthcare). This program allows obtaining the number of spots in each gel, to characterize the isoelectric point values and molecular mass, and it also allows obtaining the expression levels of the spots in the gels.

The gels were replicated three times for each sample (S2 and S3 Figs), in order to increase the reproducibility of the analysis. In each treatment, a gel with a higher correlation coefficient with the two remaining gels. All gels were juxtaposed, so the spots could be automatically recognized in all gels analyzes, and, when necessary, some manual editions were performed. The protein profiles of the gels were analyzed and compared.

### MS- based spot identification

The spots selected for identification were excised from gel 2DE and submitted to tryptic digestion as per Shevchenko *et al*. [17]. The solution containing the proteolytic digestion was fragmented through reversed-phase chromatography in a nanoAcquity UPLC (WATERS) attached to the mass spectrometer Q-Tof micro (Waters), according to Oliveira et al. [18].The obtained spectra were analyzed using software ProteinLynx v2.3 and compared against the NCBI’s database—the tool used was MASCOT MS/MS IonSearch (www.matrixscience.com), using the following parameters: digestion through trypsin enzyme, with one lost cleavage site, carbamidomethylation of cysteines (Cys) as fixed and variable modification to methionine (Met) oxidation, tolerable error of 30 ppm, mass error tolerance of 0.3 Da and 0.1 Da for fragmented ion error. After the proteins were identified, the categorization as per their ontology and function was conducted with BLAST2GO (www.blast2go.com).

### Antioxidative enzyme assays

The activities were conducted in five replicates of already-obtained samples of four-plant pools (S1 Table). The bark tissue was macerated in liquid nitrogen and homogenized in 800 μL of potassium phosphate buffer. K-P buffer was prepared by mixing 50 mmol.L^-1^ KH_2_PO_4_, and 50 mmol.L^-1^ K_2_HPO_4_ and pH was adjusted to 7.0.

Catalase (CAT) activity was dosed at 30°C with the use of a reaction buffer containing sodium phosphate at 50 mmol.L^-1^ (pH 7.0) with addition of 1 μL of the vegetable extract. The reaction was initiated through the addition of H_2_O_2_ at 30 mmol.L^-1^ and readings were conducted by calculating the absorbance decay at 240 nm for 300 s against a vegetable extract-free blank. The activity was expressed in mmol H_2_O_2_.gMF^-1^min^-1^.

Superoxide dismutase activity was determined as per Gianopolitis and Ries [19], with a few adaptations. According to that method, the activity is performed by taking into account the inhibited reduction of NBT (nitro blue tetrazolium) by the enzyme extract. The crude extract was added to the extraction buffer containing potassium phosphate buffer (50 mmol (50 mmol.L^-1^, pH 6.0), EDTA (1 mmol.L^-1^) and methionine (13mmol.L^-1^). The activity was initiated with the addition of riboflavin (1 mmol.L^-1^). The first reading was performed in the dark for 5 minutes and the second one was performed after the reaction was submitted to a 15-w fluorescent light for another 5 minutes. Both readings were performed at 560 nm. The wells were considered as blank when they did not contain crude vegetable extracts.

Guaiacol Peroxidase (GPX) activity was performed according to the methodology described by Rehem *et al*. [20], with some modifications. The activity buffer used was composed of sodium phosphate at 20 mmol L^-1^ (pH 6.0), 40 mmol L^-1^ of guaiacol and hydrogen peroxide at 0.06%. 1 μL of the previously diluted extract was added to that solution. The absorbance readings were performed at a 470 nm wavelength at 25°C. GPX activity was expressed according to the increased guaiacol consumption in μmol s^-1^ g ^-1^ of fresh biomass, from a standard curve as prepared by Rehem *et al*. [20].

The inhibiting effect of ascorbic acid (AsA) concerning guaiacol peroxidase (GPX) was performed according to Khan and Robinson [21]. The reaction was performed with 10, 20 e 30 mmol L^-1^ ascorbic acid. In this case, GPX activity can be retarded with the presence of ascorbic acid, a competing substrate.

### Statistical analyses

After the enzyme assays and the gene expression through RT-qPCR were conducted, the averages were submitted to Tukey statistical test, taking into account p values ≤ 0.05. The tests were conducted with the use of software Bio Estat v 5.3.

## Results

### Phenotypic reactions of plants to CTV

**Fig 1:**
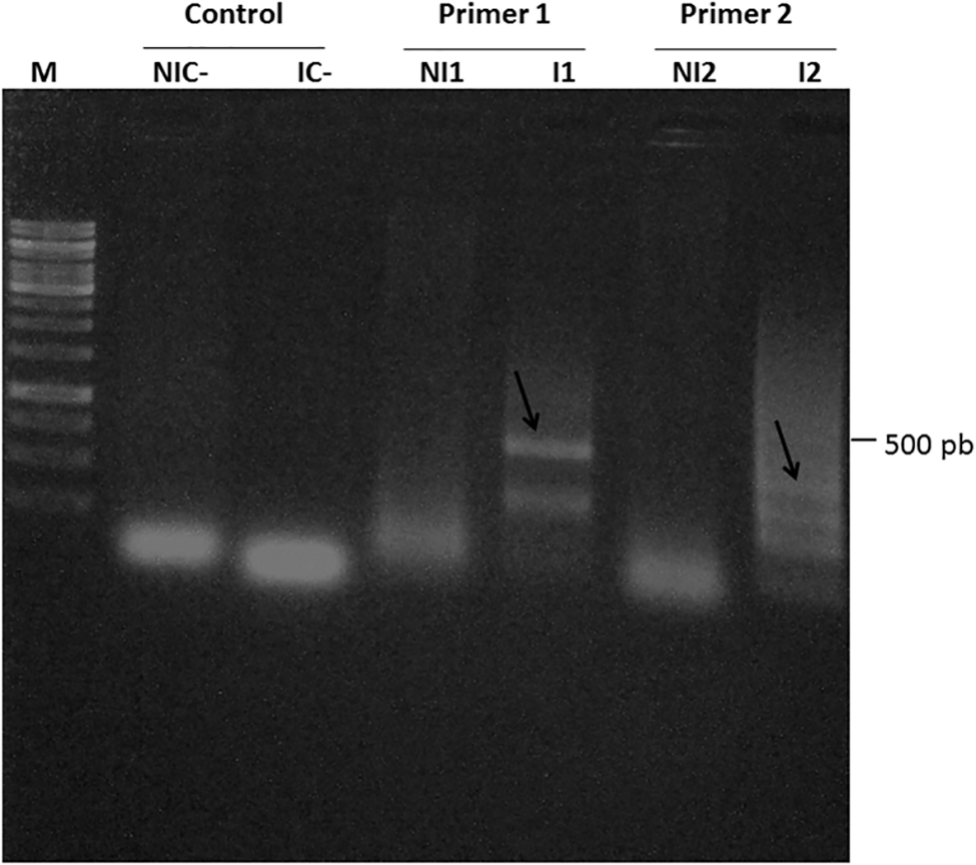
Amplification of CTV viral particles through RT-PCR in order to confirm the presence of the virus in infected samples, and to confirm its absence in non-infected samples. **M,** molecular weight marker; **NIC-** and **IC-,** negative controls of the reaction for each of the primers (1 and 2). **NI1** and **I1**, non-infected and infected samples (primer 1—CN487/489), respectively. **NI2** e **I2**, non-infected and infected samples (primer 2—CN488/491), respectively. The arrows show the amplified bands as per their expected sizes. The reaction was confirmed in a 1% agarose gel.

To check for CTV presence in infected and non-infected plants, respectively, samples were analyzed through PCR. Viral protein capsule segments were amplified through RT-PCR in infected samples, but there were no amplification in non-infected samples (Fig 1). In regards to the symptoms, according to the evaluation through the use of the rating scale, infected tree branches presented sparse stem pitting throughout their stem branches (S1 Fig).

### Differentially expressed proteins analyses and functional classifications

**Fig 2:**
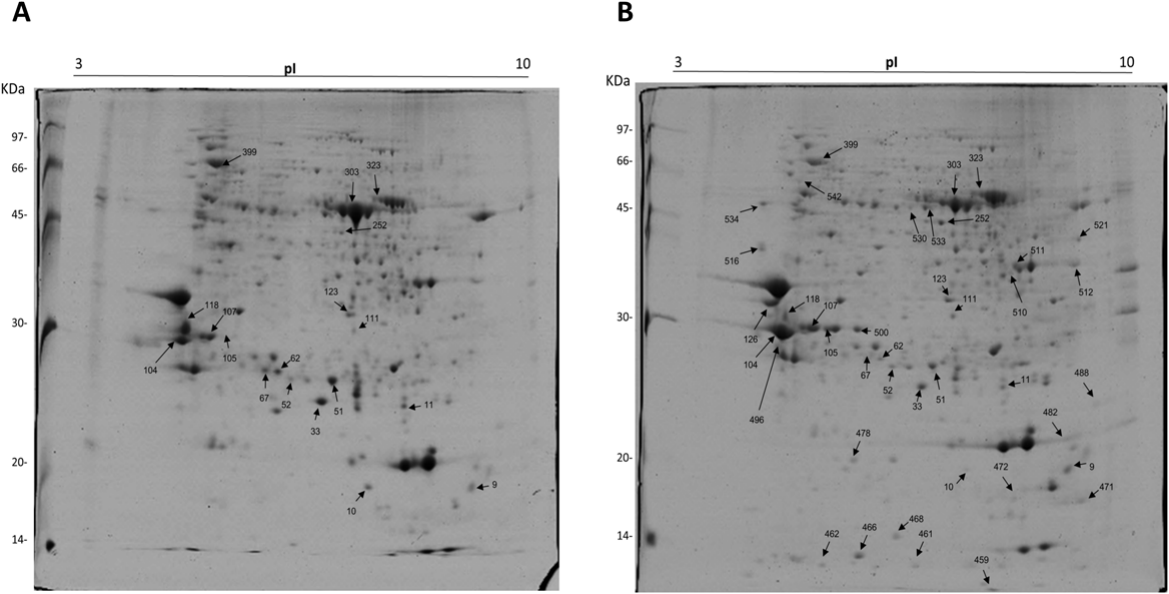
Protein Profile. Differential profile (according to the statistical analysis performed by the software) of the expression of identified proteins between non infected (A) and infected (B) treatments of “Westin” sweet orange.

**Fig 3:**
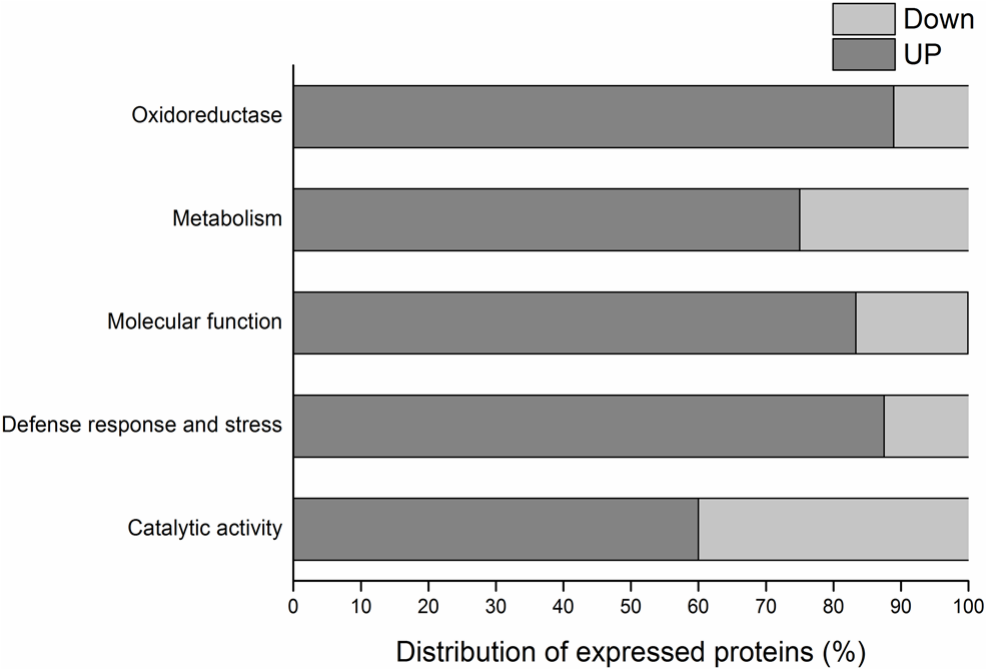
Distribution of differentially-expressed proteins of sweet orange variety “Westin” in response to *Citrus tristeza virus* (CTV), according to their expression levels. The gray segment of the bar corresponds to up-accumulated proteins and in light gray, the down-accumulated ones.

Branch bark proteins in CTV-infected and non-infected citrus plants of Westin variety were separated in 2D gels. The non-infected sample presented 604 spots total, of which 158 were exclusive; in the infected sample, a total of 618 spots were identified, 144 of which exclusive. The two treatments shared 460 spots. A total of 50 differentially-expressed spots (Fig 2A and 2B) (S2 Table) were processed, and 40 were identified through mass spectrometry. Among those, 32 were upregulated in infected samples (including 22 exclusives), and 8 were down regulated. Those spots are described in Table 2 with their corresponding proteins. Differential proteins were classified in five categories, taking into account their differential expression level (Fig 3).

**Table 2:** Differentially expressed proteins among infected and non-infected samples of “Westin” sweet orange, identified through Mass Spectrometry (ms/ms).

Spot	Accession	Specie	Protein ID	Sequence Coverage	Mowse Score	TheoreticalpI/MM (KDa)	Number of matched peptides
9	gi|157678948	*Citrus sinensis*	PS1 reaction center subunit III	29	178	9.59/15249	4
10	gi|30575572	*Citrus paradise*	HSP19 class I	32	122	9.87/6430	2
11	gi|116643152	*Citrus sinensis*	stress-related protein	62	577	5.67/17593	14
33	gi|77417707	*Citrus maxima*	SOD Superoxidedismutase	18	156	6.03/15577	3
51	gi|310772392	*Malpighia glabra*	Dehydroascorbatereductase	14	97	6.40/23725	3
52	gi|195548074	*Citrus maxima*	Ironsuperoxidedismutase	23	248	5.16/18168	4
62	gi|309774081	*Citrus sinensis*	Abscisic acid stress ripening-related protein	11	64	5.75/20038	1
67	gi|557549110	*Citrus clementina*	Carbonic Anhidrase	11	60	5.80/28628	2
104	gi|1220144	*Citrus sinensis*	Chitinase	44	567	5.06/32450	21
105	gi|1220144	*Citrus sinensis*	Chitinase	12	65	5.06/32459	2
107	gi|508699354	*Theobroma cacao*	Photosystem II subunit O-2	21	476	5.85/35364	9
111	gi|557528152	*Citrus clementina*	Xyloglucan endotransglycolilase	25	167	6.31/33283	5
118	gi|11596188	*Citrus paradise*	Lectin-related protein precursor	44	617	5.10/29300	16
123	gi|114329641	*Citrus sinensis*	ATP synthase CF1 alpha subunit	17	493	5,68/35509	9
126*	gi|1220144	*Citrus sinensis*	Chitinase	9	104	5.06/32459	1
252	gi|508717796	*Theobroma cacao*	Calreticulin 3 isoform 1	8	99	6.12/50007	2
303	gi|114329664	*Citrus sinensis*	Ribulose 1,5-bisphosphate carboxylase/oxygenase	38	1054	6.29/52999	39
323	gi|262192812	*Citrus maxima*	Catalase	29	299	6.00/38716	8
399	gi|762844	*Solanum lycopersicum*	Hsc70	7	211	5.18/71869	4
459*	gi|304565	*Citrus tristeza virus*	Coat protein	7	170	6.84/13001	1
461*	gi|304565	*Citrus tristeza virus*	Coat protein	10	113	5.37/13384	4
462*	gi|87299377	*Citrus jambhiri*	Miraculin-like protein 2	15	87	5.61/24447	3
466*	gi|2274917	*Citrus sinensis*	Cu/Zn superoxidedismutase	23	253	5.82/12777	5
468*	gi|255571035	*Ricinus communis*	Nucleoside diphosphate kinase	11	103	6.30/16301	4
471*	gi|449441230	*Cucumis sativus*	Peroxiredoxin Q, chloroplastic-like	19	59	9.70/23678	3
472*	gi|462403124	*Prunus persica*	CBS domain-containing protein CBSX3, mitochondrial-like	15	60	9.21/22744	2
478*	gi|116643152	*Citrus sinensis*	Stress-related protein	41	286	5.67/17593	4
482*	gi|119367468	*Citrus hybrid cultivar*	Miraculin-like protein 2	21	121	8.18/23610	3
488*	gi|146454508	*Sonneratia alba*	60S ribosomal protein large subunit 9	8	85	9.70/20965	1
496*	gi|151547430	*Citrus sinensis*	Cysteine protease Cp	20	183	6.25/40045	4
500*	gi|222875436	*Gossypium hirsutum*	Gibberellin 20-oxidase	2	67	6.11/43094	2
510*	gi|557532346	*Citrus clementina*	Bisphosphate aldolase cytoplasmic isozyme-like	7	95	6.96/36597	2
511*	gi|557532346	*Citrus clementina*	Fructose–bisphosphatealdolase cytoplasmic isozyme-like	45	507	6.96/36597	11
512*	gi|557536631	*Citrus clementina*	Peroxidase 12-like	25	343	8.55/38106	6
516*	gi|147790682	*Vitis vinifera*	Cysteine proteinase RD21a-like	7	97	5.10/52761	4
521*	gi|42561300	*Mycoplasma mycoides*	Prolipoprotein	1	62	9.13/83310	2
530*	gi|557522891	*Citrus clementina*	Adenosylhomocysteinase-like	5	69	5.84/53686	2
533*	gi|490329921	*Klebsiella pneumoniae*	2'-5' RNA ligase	18	66	6.34/52111	9
534*	gi|557535644	*Citrus clementina*	Calreticulin-like	18	89	4.69/41373	3
542*	gi|134101	*Ricinus communis*	Rubisco	6	98	4.77/52461	2

* Exclusives Spots from Infected Samples

The peptides were sequenced through ms/ms

Score corresponding to the coverage value, as calculated by Mascot.

Among identified proteins, 86% presented a higher expression level (up-accumulated) in the sample infected with CTV. Among them, proteins related to biotic stress in plants especially stand out, such as chitinase (spots 104 and 105). Another example of high expression level happened with the protein related to abscisic acid (spot 62). That protein presented a high expression level in plants submitted to biotic stress (CTV-infected). Some proteins involved with the redox process were identified; two of them stand out, Catalase (spot 323) and superoxide dismutase (spots 33 and 52). Catalase (CAT) presented an expression ratio of -0.08, when infected and non-infected samples were compared. In turn, superoxide dismutase (SOD) presented an expression ratio of 0.85 for spot 33 and 1.20 for spot 52. CAT expression level was reduced in infected samples. For SOD, in turn, its expression was higher in those samples.

Some proteins involved in cell function presented low expression level, among which Photosystem I Protein stands out. It acts in the photosystem reaction core (spot 9). Proteins Hsc70 (spot 399) and Hsp19 (spot 10) also presented low expression level.

**Fig 4:**
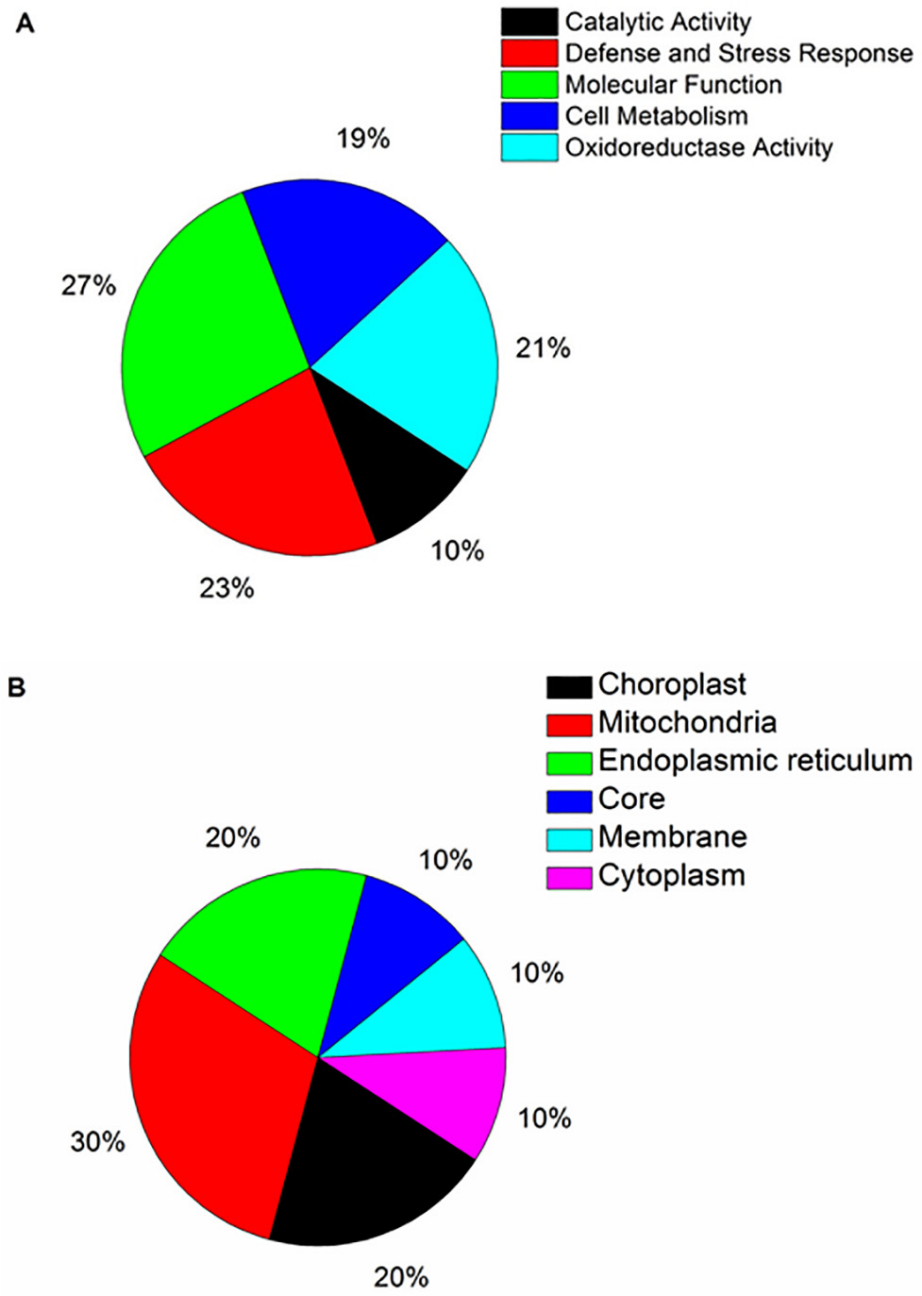
Functional categorization and cell location of identified differentially expressed proteins in infected and non-infected sweet orange variety “Westin” subjects. **A,** Functional categorization of significantly different proteins. **B,** Cell location of differentially expressed proteins.

The identified proteins were separated in five different groups according to predicted functions (Fig 4A). Most of those proteins (27%) are involved in the performance of molecular functions, 10% of them are involved in catalytic activities, 23% are involved in defense and stress responses, 19% are involved in cell metabolism, and 21% of them are involved in redox activities. Besides that, those proteins were also separated by taking into account their locations in cell organelles (Fig 4B). Most proteins are located in the mitochondria (30%), 20% are located in the endoplasmic reticulum, 20% is located in the chloroplasts. The proteins located in the nucleus, in the cytoplasm, and in the membranes total 10% in each category.

A viral protein was identified in spots 459 and 461. For spot 459, three peptides were sequenced through mass spectrometry, totaling 51 amino acids. For spot 461, a peptide was found that was common to the one found in spot 459, and it contained a total of 19 amino acids, corresponding to the central region of a viral capsule protein with 223 amino acids. The 51 amino acids cover 22.8% of the viral protein capsule with 100% identity.

### Antioxidant enzyme activities

**Fig 5:**
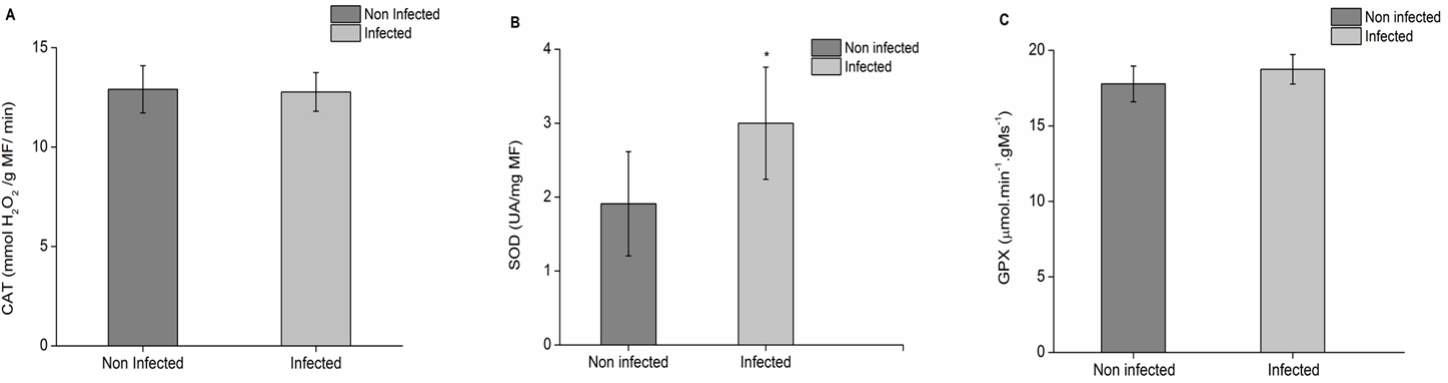
Enzyme activity in “Westin” sweet oranges infected and non-infected with CTV. **A,** Catalase **B,** Superoxide Dismutase **C,** Guaiacol Peroxidase. The vertical columns indicate the average absorbance values (n = 4). The bars above the columns represent the standard error of averages. The asterisk (*) indicates that the values presented a significant difference through Tukey test, taking into account p ≤ 0.05.

**Fig 6:**
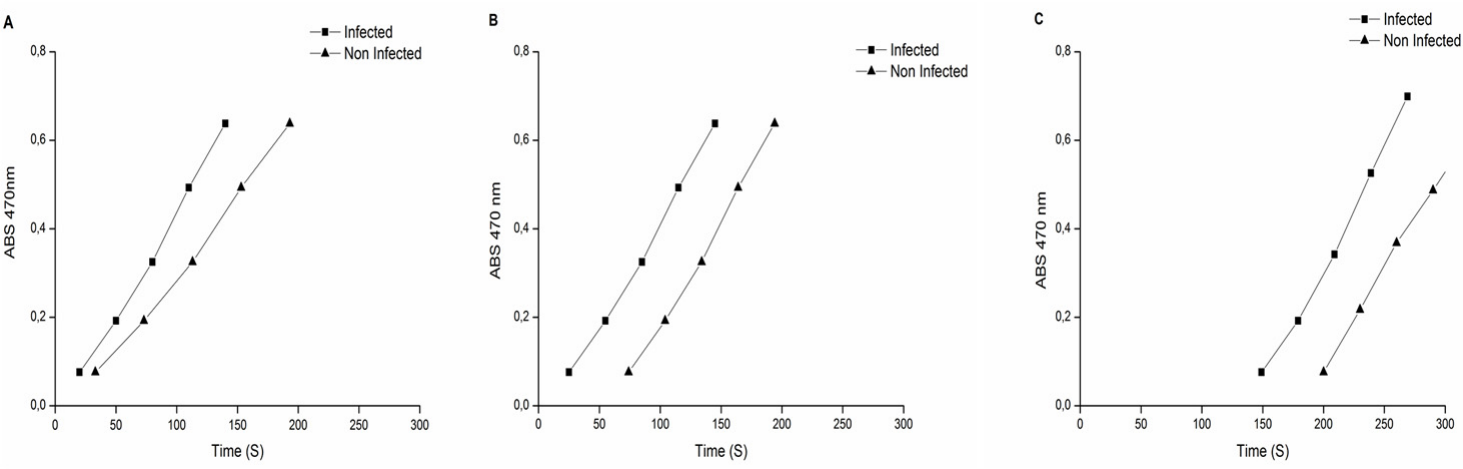
Peroxidase activity in “Westin” sweet orange infected and non-infected with CTV, with the use of ascorbic acid and guaiacol as electron donors. **A, B, and C** AsA—exhaustion times in the reaction at 10 mmol.L^-1^, 20 mmol.L^-1^ and 30 mmol.L^-1^, respectively. The arrows indicate the times at which GPX activity was started.

The activity of some enzymes with a protection potential against the oxidative stress was analyzed in the two treatments, and some significant differences were observed. From the analyzed enzymes, only CAT presented an activity which was 0.2x higher in the non-infected sample, but the difference was not significant. SOD and GPX presented a higher activity in the infected sample, 2x and 1x higher, respectively (Fig 5A–5C). In turn, in regards to APX activity, which was measured as per the preferential use of substrates, in 10 mmol.L^-1^ of AsA (Fig 6A), the elevation of ABS470 started at 33 seconds for non-infected samples. For the infected ones, it started at 20 seconds. In a medium containing 20 mmol.L^-1^ of AsA (Fig 6B), the enzyme activity with guaiacol initiated at 74 seconds for non-infected samples and at 25 seconds for infected samples. Finally, with a concentration of 30 mmol.L^-1^ of AsA (Fig 6C) at 470 nm, the enzyme activity was observed in non-infected samples at 200 seconds and at 149 seconds, approximately, in infected samples. In all concentrations, the use of guaiacol as an electron donor was first started in CTV-infected samples.

### RT-qPCR

**Fig 7:**
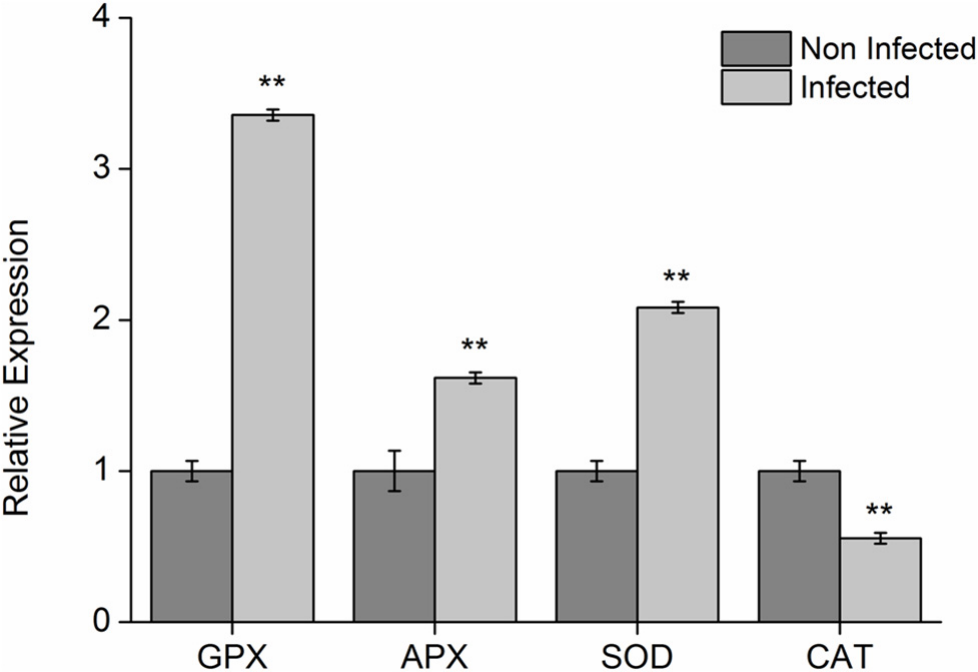
Analysis of the accumulation of transcripts of the genes encoding enzymes involved in oxidative stress in sweet orange variety “Westin” plants infected and non-infected with CTV. The columns marked with ** indicate that the values among treatments presented significant differences through Tukey test (p ≤ 0.001. The vertical columns indicate the average values of Cts which were calculated through method 2^-ΔΔCt^ (LIVAK E SCHMITTGEN, 2001), the bars above the columns indicate the standard error of averages.

The relative expression levels of the genes of some of the enzymes involved in the oxidative stress were evaluated. The expression levels of the genes which code those enzymes were higher for CTV-infected plants. APX accumulation in the infected samples increased twofold as compared to the non-infected samples. The accumulation of the transcript which codes GPX was almost three times as high in the infected samples. The accumulation of SOD transcript was twice as high in the infected samples, whereas the expression of CAT gene was reduced in 0.5 times in the infected samples (Fig 7).

## Discussion

### Proteomic analyses

The viral infection promotes many changes in the protein profile of plants [22]. Our results with *Citrus tristeza virus*-infected and non-infected samples of “Westin” sweet orange showed interesting alterations. In this case, besides the presence of exclusive spots for each sample, 23 spot were also detected, with different expression levels, which indicated the induced and repressed accumulation of proteins in the virus presence. Similarly, Giribaldi *et al*. [23] analyzed proteomic alterations caused by viruses which were restricted to *Vitis vinifera* phloem, and found 400 spots in total, of which only 19 had differential expression levels. In this study, no exclusive spots were identified.

The differences found in samples which were either infected by a pathogenic virus or not may be related to stress and defense responses, as they play an important role during cell infection processes. The proteomic analysis of the interaction between plants which are resistant and susceptible to sugarcane virus showed that a great part of identified proteins was mainly related to stress and defense [22], but no exclusive spots were identified, only two differentially expressed spots. On the other hand, Cantú *et al*. [7], when performing a study with citrus plant barks which were both affected and unaffected by “Sudden Death in Citrus”, observed a difference in protein patterns of analyzed samples. The authors found exclusive proteins in infected and non-infected samples, but found no common proteins with significant differential expression levels.

### Proteins involved with catalytic activity, photosynthesis and metabolism

Most studies with proteomic analyses of plants infected by microorganisms find reduced accumulation of proteins which are involved in photosynthesis [24,25]. According to Monavarfeshani *et al*. [26], it happens because when a plant is submitted to the attack of a virus or of any other microorganism, it tends to first dedicate to the production of compounds related to the response to the stress caused by that attack, and those rates only come back to normal when the micro-organism stops growing. It is also important to point out that a pathogen such as CTV, lodged in the phloem, may affect the flow of photoassimilates and, hence, inhibit the photosynthesis. In that study, RuBisCO (spot 303) showed an expression level which was significantly reduced (down-accumulated). *1*,*5-bisphosphate carboxilase oxigenase* (RuBisCO) is an important enzyme, due to the fact it helps plants assimilate carbon dioxide and catalyzes the reaction which breaks CO_2_ and facilitates its assimilation in its organic form [27]. The difference in the accumulation of the RuBisco protein, found in this study, suggests that its expression is affected due to the stress which is caused by the infected status with the virus. Grapefruits infected with *Candidatus* Liberibacter asiaticus (Las), a bacterium that is restricted to citrus phloem, which causes the *Huanglongbing* (HLB) disease, also presented a reduced accumulation of RuBisCO [28].

In transgenic tomato leaves infected with the *Cucumber mosaic virus*- CMV, most proteins which presented different expression levels were related to photosynthesis. Among them, RuBisCO, which presented itself as down-accumulated [29].

The protein of sub-unit II of photosystem reaction center I (PSI) (spot 9) and the protein of sub-unit O2 of photosystem II (PSII) (spot 107) were found to be down-accumulated in infected plants. Those proteins are related to photosynthesis reaction sites, and they are responsible for the conversion of luminous energy into chemical energy [30]. Although the accumulation level of those proteins is expected to be reduced, as a defense response of the plant against pathogens, the opposite happened. In this case, the presence of the virus may be inducing the expression of those proteins for their own benefit. Consequently, the photosynthetic yield of those plants is not affected, which keeps the plant alive [31]. On the other hand, the increase of some proteins may take place, so the plant can make up for the reduction of other proteins. Besides that, the increase of a protein not always implies the increase of a process [22]. *C*. *sinensis* plants infected with *Xanthomonas axonopodis* were studied with the use of classic proteomics as a tool [31]. In plants which were infected with recombinant bacteria, the symptoms were far more severe, and the accumulation of proteins related to photosynthesis increased, instead of decreasing. It suggested a molecular mimicry, with the pathogen inducing the expression of those proteins, and consequently increasing photosynthesis rates for its own benefit. More specific studies may clarify whether something similar happens due to CTV action in citrus plants.

Regarding the proteins that are involved in cell metabolism, ATP synthase (spot 123) was identified to be up-accumulated in CTV-infected plants. It is a key enzyme for ATP production, as it catalyzes ADP phosphorylation [32]. Several important enzymes, involved with energy production and cell metabolism generally are found to have reduced accumulation level in processes involving the host/pathogen interaction, as it happens with plants infected with bacteria, for example [33], which indicates a likely response from the microorganism virulence, and consequently, a positive feedback for its benefit.

### The presence of proteins related to Biotic Stress

The infection of sweet oranges by CTV resulted in an increased accumulation of some proteins involved with stress and defense responses against pathogens. The protein related to stress (spot 11) was found to be up-accumulated after virus infection. That protein is activated when the plant is submitted to pathogen attacks [33]. Other proteins involved with stress responses were highlighted in this study, as they were found to be up-accumulated in infected samples. Among those proteins, the one related to abscisic acid (spot 62), to chitinase (spots 104 and 105), to Hsp19 protein (Spot 10), and Lectin-precursor protein (spot 118).

An up-accumulated protein related to ABA (abscisic acid) stress was identified, and therefore, the action of that hormone may have been affected by the virus presence. ABA is a phytohormone which is present in all plants. It is directly related to retarded or accelerated development of vegetables, depending on the environmental conditions that they are submitted to, especially in stress situations like drought stress [34]. ABA is also related to biotic stress, and it operates mainly in responses to both kinds of stress, once the responses against biotic and abiotic stresses may take place simultaneously [35]. In maize infected with sugarcane mosaic virus, that protein was induced, although its biological role is not very known yet [22]. Its super expression in transgenic tobacco increased the tolerance to salinity [36].

Two chitinases were identified in spots 104 and 105 (Fig 3) as up-accumulated. Chitinase regulation is induced by several factors, including pathogen attacks and abiotic stresses involving heavy metals or vegetable hormones, and they are responsible for the hydrolysis of chitin polymer, which is present in several organisms [37]. Those proteins, as well as others related to stress responses play an important role against specific pathogens [38]. In plants infected with the pepper virus, four different types of up-accumulated chitinases were identified, with acid and basic isoelectric points [39]. Chitinase induction is generally present with the induction of other stress-related proteins (PR) [37].

The lectin precursor protein (spot 118) was found to be up-accumulated (2x) in the infected sample. It highlights the fact that the virus is restricted to the phloem, and it consequently causes tissue damage. Lectins belong to a protein group which plays several roles in vegetable cells, and they may be found in several organisms besides plants. One of their main roles is related to the differentiation of vascular tissues, and their ability to bind to several sugar molecules. Those characteristics may be related to phloem regeneration as a response against the attack of microorganisms or insects which may feed from that tissue [40]. The ability to bind to several different molecules may be one of the reasons for the expression of this protein during a viral infection process, and it may interact with the viral RNA molecule, preventing its action and consequently preventing the infection progression [41]. A recombinant lectin from *Musa paradisiaca* exhibited effects on *Tobacco mosaic virus* (TMV). It is may prevent TMV infection and maintain relatively normal physiological parameters such as chlorophyll content, photosynthetic rates and enhanced antioxidant activities [42]. That was also proved in bacteria by Kim *et al*. [43], who showed that the presence of a lectin-related protein resulted in the blockage of the *C*. *Liberibacter asiaticus* bacterium translocation, infecting sweet orange *C*. *sinensis*.

### Proteins related to Oxidative stress

Some proteins related to the presence of oxidative stress in plants were identified. Among those enzymes, SOD, CAT and the peroxidases stand out. Superoxide dismutase (spot 33) and iron-dependent superoxide dismutase (spot 52) were shown to be up-accumulated in infected samples. In turn, catalase (spot 323) was found to be down-accumulated. This may have occurred because probably the damage was localized in chloroplasts whereas the CAT enzyme is absent from chloroplasts and is localized in peroxisomes [44]. Regarding aerobic organisms, the production of reactive oxygen species (ROS) invariably happens, and that may be intensified when the organism is submitted to a kind of stress. Some enzymes are recruited to attack the ROS and regulate cell function [45].

SOD is an enzyme which has the ability to catalyze O_2_˙ˉ dismutation, producing O_2_ and H_2_O_2_ [45]. In turn, catalase is responsible for decomposing H_2_O_2_ into water and molecular oxygen (O2). Those enzymes are related to the infection and to oxidative stress tolerance in plants [46,47]. SOD enzyme activity was higher in infected samples, as well as the expression level of the gene which codes it. In turn, CAT activity presented differences, but the gene expression level was higher in the infected treatment. According to Govrin and Levine [48], the repression of enzymes related to peroxide decomposition may facilitate the development and the virulence of pathogens. Besides that, such result also suggests that, so far, the responses to oxidative stress are more complex than a simple increase or decrease in the expression level of antioxidant enzymes [22].

The peroxidase activity was evaluated in regards to the use of two substrates, guaiacol and ascorbate. Those two compounds act as electron-donor substrates for the degradation of H_2_O_2_ by ascorbate peroxidase (APX) and guaiacol peroxidase (GPX) enzymes, generating H_2_O [49]. In all ascorbate concentrations used, the depletion of that substrate and the starting of the use of guaiacol in the reaction medium first started in Tristeza virus-infected samples, indicating that APX-type peroxidases predominate in infected plants. AsA presence in meristematic tissues was observed to prevent the peroxide coming from the extracellular medium from entering, until it has been fully consumed inside the cell. Besides that, AsA presence inhibits guaiacol peroxidase activity [50].

### Proteins related with the growth and metabolism of viruses

A virus capsule-related protein was detected, and it suggested increased titration of the virus in Tristeza virus-infected sweet oranges. The estimated masses for spots 459 and 461 are 13.001 and 13.384 kDA, respectively, along with the presence of the central peptide, indicating that both spots resulted from proteolytic cleavages in N and C terminuses of the protein in the capsule (gi|304565), whose total estimated mass is 24.930 KDa. 2D-PAGEs reveal that the sample collection and extraction methods were efficient to preserve the integrity of proteins. The spots which correspond to the protein in the capsule may result from the action of the plant defense proteases. The detection of that protein which composes the viral protein capsule may be considered as compatible with the fluting degree which was observed in sampled plant branches. Those results suggest that “Westin” sweet orange may not be tolerant to the virus, which highlights that the infection is reaching levels which may reduce productivity and consequently cause economic losses. *Nicotiana spp*. and *Arabidopsis thaliana* plants also presented a protein which is related to the protein in the capsule of an unknown virus [51], as well as *N*. *bentamiana* plants infected with *Pepper mild mottle tobamovirus* [52], but Cantú *et al*. [7] and Wu *et al*. [22] could not identify any pathogen protein in their studies.

The viruses invariably have an intracellular nature. Thus, their proteins and nucleic acids can interact and influence the protein activity of the hosts after their attack. Those viral proteins build up, and, many times, they suppress the defense of their hosts, which, in turn, suffer from systemic infections as those accumulated proteins having functions which facilitate the invasion throughout the infected plants [53].

## Conclusions

In this study, it was demonstrated that sweet orange variety “Westin” responds to the presence of CTV with variations in its protein profile, and in the expression of specific genes. The CTV infection induced an increased oxidative stress, as the activity of antioxidant enzymes was higher in the infected sample, as well as the expression of genes which correspond to SOD, GPX, and APX. “Westin” variety uses AsA as its preferential electron donor. According to the fluting detected on infected plants, along with the detection of proteins from CTV capsule, which was detected in two-dimensional gels, the tolerance of “Westin” variety to *Citrus tristeza virus* is made questionable. Our results suggest that these plants contain proteases which act in the defense by cleaving proteins which compose the viral capsule.

## Supporting Information

S1 Fig Symptom of stem pitting. Infected branches with the symptoms characterized by the presence of sparse stem pitting throughout their stems. The arrows show the presence of stem pitting.
(PDF)

S2 Fig Replicate of two dimensional gel of non-infected sample. 2D gels of non- infected samples to increase the reproducibility of the analysis.
(PDF)

S3 Fig Replicate of two dimensional gel of infected sample. 2D gels of infected samples to increase the reproducibility of the analysis.
(PDF)

S1 Table Dataset underlying the results. Spreadsheet containing all raw data about the Enzyme Activities and RT-qPCR.
(XLSX)

S2 Table Identified Proteins. Table containing additional information about the proteins that were identified and ms/ms data.
(PDF)
