# Supplementary figures and images for: Citrus tristeza virus (CTV) Causing Proteomic and Enzymatic Changes in Sweet Orange Variety “Westin”
Source: PLoS One. 2015 Jul 24;10(7):e0130950. doi: 10.1371/journal.pone.0130950 (PMC4514840; doi:10.1371/journal.pone.0130950)

**S1 Fig. Symptom of stem pitting.**

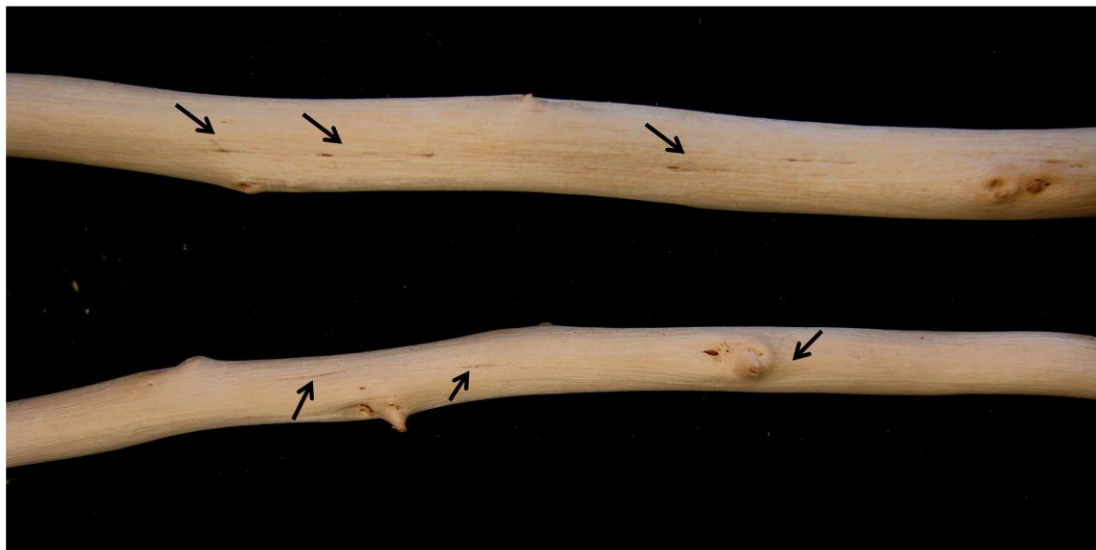

Supplement: S1 Fig — Infected branches with the symptoms characterized by the presence of sparse stem pitting throughout their stems. The arrows show the presence of stem pitting. (PDF) [file pone.0130950.s001.pdf]

**S2 Fig. Replicate of two dimensional gel of uninfected sample.**

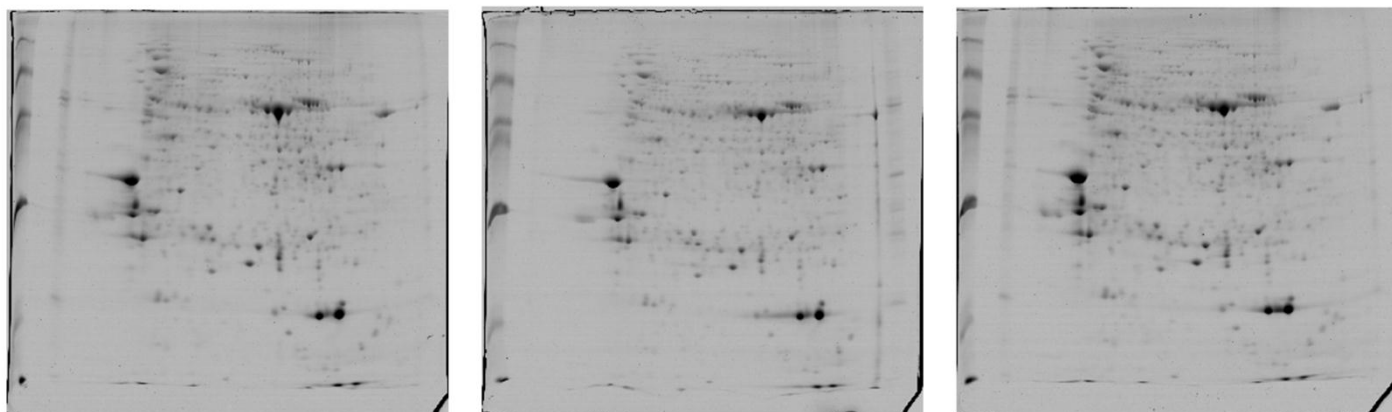

Supplement: S2 Fig — 2D gels of non- infected samples to increase the reproducibility of the analysis. (PDF) [file pone.0130950.s002.pdf]

**S3 Fig. Replicate of two dimensional gel of infected sample.**

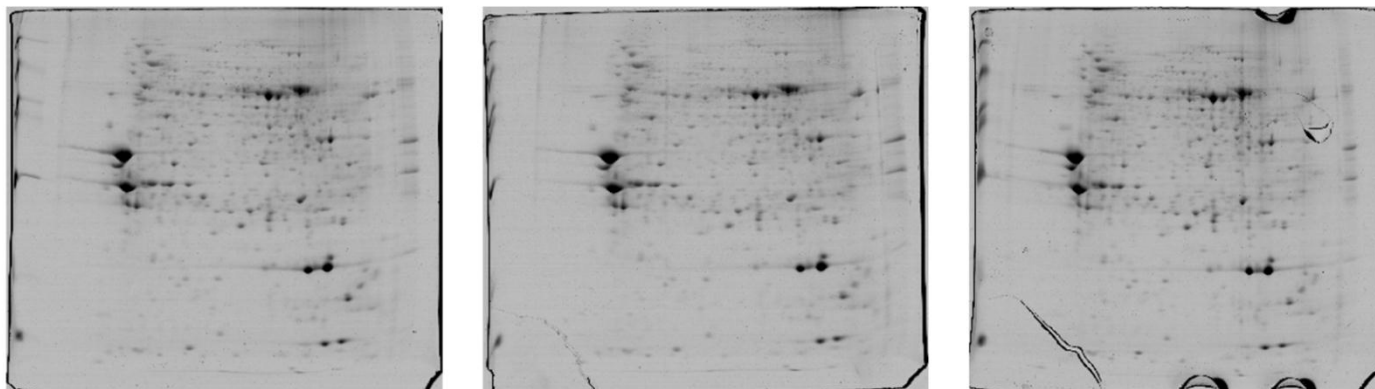

Supplement: S3 Fig — 2D gels of infected samples to increase the reproducibility of the analysis. (PDF) [file pone.0130950.s003.pdf]
