# Supplementary material for: Citrus tristeza virus (CTV) Causing Proteomic and Enzymatic Changes in Sweet Orange Variety “Westin”
Source: PLoS One. 2015 Jul 24;10(7):e0130950. doi: 10.1371/journal.pone.0130950 (PMC4514840; doi:10.1371/journal.pone.0130950)
Supplement: S2 Table — Table containing additional information about the proteins that were identified and ms/ms data. (PDF) [file pone.0130950.s005.pdf]

Spot	Protein / organism / accession	MM	pI	Coverage/ Mowse Score	Peptides/ charge
9	PS1 reaction center subunit III [Citrus sinensis]<br><br>gi|157678948	15249	9.59	29% / 178	R.ESYWYNGIGSVVAVDQDPK.S(2+)<br><br>R.FNKVNYANVSTNNYALDEIEEVK.- (3+)<br><br>R.FNKVNYANVSTNNYALDEIEEVK.- (3+)<br><br>K.VNYANVSTNNYALDEIEEVK.- (2+)
10	HSP19 class I, partial [Citrus x paradisi]<br><br>gi|30575572	6430	9.87	32% / 122	K.ASMENGVLTVTVPK.Q + Oxidation (M)(2+)<br><br>K.ASMENGVLTVTVPKQEEK.K + Oxidation (M)(2+)
11	PREDICTED: stress-related protein/Citrus sinensis/<br>gi|116643152	17593	5.67	62%/577	M.GVLTLNAEETSTLPPEK.L (2+)<br><br>M.GVLTLNAEETSTLPPEK.L (2+)<br><br>M.GVLTLNAEETSTLPPEKLFK.L (2+)<br><br>M.GVLTLNAEETSTLPPEKLFK.L(3+)<br><br>K.LFVLHFDTLLPK.V (2+)<br><br>K.NVELISGDGGPGSIK.K (2+)<br><br>K.NVELISGDGGPGSIKK.F (2+)<br><br>K.NVELISGDGGPGSIKK.F (2+)

					K.FNFVEGADWK.Y (2+)<br>R.VDALDKENK.I (2+)<br>K.STVVIKFYPKPGAEIK.E (2+)<br>K.FYPKPGAEIKEEQVK.G (3+)<br>K.FYPKPGAEIKEEQVK.G (2+)<br>K.ALEAYALANPNAV.- (2+)
33	SOD Superóxido dismutase / gi|77417707/Citrus maxima	15577	6.03	18% / 156	K.AVEQLFQALNK.V (2+)<br>R.LVVETTANQDPLVTK.A (2+)<br>R.LVVETTANQDPLVTK.A (2+)
51	dehydroascorbatereductase / gi|310772392/Malpighia glabra	23725	6.40	14%/97	R.HLFDLSNKPQWFLEISPEGK.V (3+)<br>R.VTAVDLSLAPK.L (2+)<br>R.VTAVDLSLAPK.L (2+)
52	Iron superoxide dismutase /gi|195548074/ [Citrus maxima]	18168	5.16	23%/ 248	K.QIVGTELGDGK.S (2+)<br>K.SLEDVVIASYNK.G (2+)<br>R.ADVANAVNPLPSEK.D (2+)<br>R.ADVANAVNPLPSEKDK.S (2+)

62	abscisic acid stress ripening-related protein/ gi|309774081/[Citrus sinensis]	20038	5.75	11%/64	K.HLEHLGELGTAGAGAFALLEK.H(3+)
67	Carbonic Anhidrase / gi|557549110/ Citrus clementina	28628	5.80	11%60	R.NVANIVPPYDQTK.Y<br>K.EAVNVSLSNLLTYPFVR.E
104	Chitinase / gi|1220144 /Citrus sinensis	32459	5.06	44% /567	K.IISREMFDDLLEYR.N + Oxidation (M)(2+)<br>R.EMFDDLLEYR.N(2+)<br>R.EMFDDLLEYR.N + Oxidation (M) (2+)<br>R.EMFDDLLEYR.N + Oxidation (M) (2+)<br>R.EMFDDLLEYRNDER.C(2+)<br>R.GPIQLSWNYNYLR.C(2+)<br>R.GPIQLSWNYNYLR.C(2+)<br>R.CGEGLGLGEELLNNPDLLATDPVLSFK.S(2+)<br>R.CGEGLGLGEELLNNPDLLATDPVLSFK.S (3+)<br>R.CGEGLGLGEELLNNPDLLATDPVLSFK.S(3+)<br>R.CGEGLGLGEELLNNPDLLATDPVLSFK.S(3+)<br>R.CGEGLGLGEELLNNPDLLATDPVLSFK.S(3+)<br>R.CGEGLGLGEELLNNPDLLATDPVLSFK.S(3+)

					K.SAIWFWMTAQPPKPSCHEVIIDEWKPSANDVNAGR.L(5+)<br>K.SAIWFWMTAQPPKPSCHEVIIDEWKPSANDVNAGR.L + Oxidation (M)(5+)<br>R.LPGYGLTTNIINGGIECGQGGNAAVR.N(3+)<br>R.LPGYGLTTNIINGGIECGQGGNAAVR.N(3+)<br>R.LPGYGLTTNIINGGIECGQGGNAAVR.N(3+)<br>R.LPGYGLTTNIINGGIECGQGGNAAVR.N(3+)<br>R.LPGYGLTTNIINGGIECGQGGNAAVR.N(3+)<br>R.NRIGFFTTFCGK.F(2+)
105	Chitinase/ gi|1220144 / Citrussinensis	32459	5.06	12%/ 65	R.EMFDDLLEYR.N + Oxidation (M)(2+)<br>R.CGEGLGLGEELLNNPDLLATDPVLSFK.S(3+)
107	Photosystem II subunit O-2<br>gi|508699354<br>Theobromacacao	35364	5.85	21% /476	K.RLTYDEIQSK.T(2+)<br>R.LTYDEIQSK.T(2+)<br>K.GTGTANQCPTIDGGVDSFAFKPGK.Y(3+)<br>K.GTGTANQCPTIDGGVDSFAFKPGK.Y(3+)<br>K.GTGTANQCPTIDGGVDSFAFKPGK.Y(3+)

					K.GTGTANQCPTIDGGVDSFAFKPGK.Y(3+)<br><br>K.KFCLEPTSFTVK.A(2+)<br><br>K.DGIDYAAVTVQLPGGER.V(2+)<br><br>R.VPFLFTIK.Q(2+)
111	Xyloglucan endotransglycolilase /gi|557528152 / Citrus clementine	33283	6.31	17%167	R.AIQLVLDQNSGCGFASK.R<br>R.AIQLVLDQNSGCGFASKR.Q<br>R.APFPMNQPMGVYSTLWEADDWATR.G + Oxidation (M)<br>K.APFYAYYR.D<br>K.APFYAYYR.D
118	lectin-related protein precursor [Citrus x paradisi] gi|11596188	29300	5.10	44%617	K.TLICYGAIESSGALSITPGPPPNLPIRK.V<br>K.TLICYGAIESSGALSITPGPPPNLPIRK.V<br>K.TLICYGAIESSGALSITPGPPPNLPIRK.V<br>R.SFIDTTITIK.I<br>R.AGDGMTFIFASDK.N<br>R.AGDGMTFIFASDK.N + Oxidation (M)<br>R.AGDGMTFIFASDK.N + Oxidation (M)<br>R.AGDGMTFIFASDK.N + Oxidation (M)<br>R.AGDGMTFIFASDKNGPSAK.G<br>R.AGDGMTFIFASDKNGPSAK.G<br>R.AGDGMTFIFASDKNGPSAK.G + Oxidation (M)<br>R.AGDGMTFIFASDKNGPSAK.G + Oxidation (M)<br>R.AGDGMTFIFASDKNGPSAK.G + Oxidation (M)<br>K.GVGEYLGLQSSPGDKFPPLAVELDTCLNK.N

					K.FPPLAVELDTCLNK.N<br>K.NLNDPDDNHIGIDINGIESNPVNSLLDVDLKSGR.A
123	ATP synthase CF1 alpha subunit [Citrus sinensis]gi|114329641	55509	5.09	17% / 493	K.ADEISNIIR.E(2+)<br>K.IVNIGTVLQVGDGIAR.I(2+)<br>K.IVNIGTVLQVGDGIAR.I(2+)<br>K.IAQIPVSEAYLGR.V(2+)<br>R.VINALAKPIDGR.G(2+)<br>R.LIESPAPGIISR.R(2+)<br>R.LIESPAPGIISR.R(2+)<br>K.ASSVAQVVNTFQER.G(2+)<br>R.EAYPGDVFYLHSR.L(2+)
126	Chitinase/ gi|1220144 / Citrussinensis	32459	5.06	9% / 104	R.CGEGLGLGEELLNNPDLLATDPVLSFK.S(3+)
252	Calreticulin3isoform 1<br>gi|508717796/ Theobromacacao	50007	6.12	8% 99	R.FEQDIECGGGYIK.L(2+)<br>K.IPYIDNPEFEDDPDLYVLKPIK.Y(3+)
303	ribulose 1,5-bisphosphate carboxylase/oxygenase large subunit [Citrus sinensis]<br>gi|114329664	52999	6.29	38%/ 1054	K.LTYYTPDYVTK.D<br>K.LTYYTPDYVTK.D<br>K.LTYYTPDYVTKDTDILAAFR.V<br>K.LTYYTPDYVTKDTDILAAFR.V<br>K.DTDILAAFR.V<br>R.VTPQPGVPPEEAGAAVAAESSTGTWTAVWTDGLTSLD

					R.Y<br>R.VTPQPGVPPEEAGAAVAAESSTGTWTAVWTDGLTSLDR.Y<br>R.VTPQPGVPPEEAGAAVAAESSTGTWTAVWTDGLTSLDR.Y<br>R.VTPQPGVPPEEAGAAVAAESSTGTWTAVWTDGLTSLDR.Y<br>R.VTPQPGVPPEEAGAAVAAESSTGTWTAVWTDGLTSLDR.Y<br>R.VTPQPGVPPEEAGAAVAAESSTGTWTAVWTDGLTSLDR.Y<br>R.VTPQPGVPPEEAGAAVAAESSTGTWTAVWTDGLTSLDR.Y<br>R.LEDLRIPPAYTK.T<br>K.TFQGPPHGIQVER.D<br>K.TFQGPPHGIQVER.D<br>K.TFQGPPHGIQVER.D<br>K.TFQGPPHGIQVER.D<br>K.TFQGPPHGIQVER.D

					K.TFQGPPHGIQVER.D<br>K.TFQGPPHGIQVER.D<br>R.AVYECLR.G<br>R.GGLDFTKDDENVNSQPFMR.W<br>R.GGLDFTKDDENVNSQPFMR.W<br>R.GGLDFTKDDENVNSQPFMR.W<br>R.GGLDFTKDDENVNSQPFMR.W + Oxidation (M)<br>R.GGLDFTKDDENVNSQPFMR.W + Oxidation (M)<br>K.DDENVNSQPFMR.W + Oxidation (M)<br>K.DDENVNSQPFMR.W + Oxidation (M)<br>R.DNGLLLHIHR.A<br>R.AMHAVIDR.Q + Oxidation (M)<br>R.LSGGDHIHAGTVVGK.L<br>R.LSGGDHIHAGTVVGK.L<br>R.LSGGDHIHAGTVVGK.L<br>R.DITLGFVDLLR.D

					R.EASKWSPELAAACEVWK.S<br>K.WSPELAAACEVWK.S<br>K.SIKFEFAAMDTL.-<br>K.SIKFEFAAMDTL.- + Oxidation (M)<br>K.SIKFEFAAMDTL.- + Oxidation (M)
323	catalase<br>Citrusmaxima<br>gi|262192812	38716	6.00	29% /299	K.GFFECTHDISHLTCADLFR.A(3+)<br>R.APGVQTPVIVR.F(2+)<br>R.HMEGFGVQTFTLVNK.N(2+)<br>R.HMEGFGVQTFTLVNK.N + Oxidation (M)(2+)<br>K.VGGSNHSHATQDLYDSIAAGNYPEWK.L(3+)<br>K.WWPEDIIPLQPVGR.L(2+)<br>R.LGPNYLMLPVNAPK.C + Oxidation (M)(2+)<br>R.LGPNYLMLPVNAPK.C + Oxidation (M)(2+)
399	Hsc70 [Solanum lycopersicum]<br>gi|762844	71869	5.18	7% / 211	K.NQVAMNPTNTVFDAK.R + Oxidation (M)(2+)<br>K.NAVVTVPAYFNDSQR.Q(2+)<br>R.IINEPTAAAIAYGLDK.K(2+)

					R.IINEPTAAAIAYGLDKK.I(2+)
459	coat protein/ [Citrus tristeza virus]/gi|304565	13001	6.84	7%/170	K.SSSLQSDDDTTGITYTR.E(2+)
462	miraculin-like protein 2<br>gi|87299377 Citrus jambhiri	24447	5.61	15%/ 87	K.IVHCPSVCESCVK.L(2+)<br>R.LVLVRDDEPAFPVVLIPATER.S(2+)<br>R.DDEPAFPVVLIPATER.S(2+)
466	Cu/Zn superoxidedismutase<br>gi|2274917<br>Citrussinensis	12777	5.82	23% / 253	R.KGTVSFSVEGSGPTTVK.G(2+)<br>K.GTVSFSVEGSGPTTVK.G(2+)<br>K.GTVSFSVEGSGPTTVK.G(2+)<br>K.TIPLSGTNSVIGR.G(2+)<br>K.TIPLSGTNSVIGR.G(2+)
468	nucleosidediphosphate kinase, putative<br>gi|255571035<br>Ricinuscommunis	16301	6.30	11%/103	R.TIIGATNPAQSAPGTIR.G(2+)<br>R.TIIGATNPAQSAPGTIR.G(2+)<br>R.TIIGATNPAQSAPGTIR.G(2+)<br>R.TIIGATNPAQSAPGTIR.G(2+)
471	PREDICTED: peroxiredoxin Q, chloroplastic-like [Cucumissativus]gi|449441230	23678	9.70	19% / 59	K.GKPVVVYFYPADETPGCTK.Q(3+)<br>K.GKPVVVYFYPADETPGCTK.Q(2+)

					R.QTYVLDKNGVVQLIYNNQFQPEK.H(3+)
472	hypothetical protein PRUPE_ppa011584mg [Prunuspersica]gi|462403124	22744	9.21	15%/ 60	K.SMTQHNVGALVVVKPGEQK.S + Oxidation (M)(3+)<br>R.GMIGMVSIGDVVR.A + 2 Oxidation (M)(2+)
473	hypothetical protein CICLE_v10029454mg [Citrus clementina] / gi|557525674	17580	6.40	9% 65	K.SVHDFVVEVVEGDAR.N
476	cyclophilin [Citrussinensis]<br>gi|260401128	18310	8.70	72%/ 599	K.VFFDMTVGGQPAGR.I + Oxidation (M)<br>K.VFFDMTVGGQPAGR.I + Oxidation (M)<br>R.IVMELFADVTPR.T<br>R.IVMELFADVTPR.T + Oxidation (M)<br>R.IVMELFADVTPR.T + Oxidation (M)<br>R.IVMELFADVTPR.T + Oxidation (M)<br>R.VIPGFMCQGGDFTAGNGTGGESIYGSK.F + Oxidation (M)<br>K.FADENFVKK.H<br>K.KHTGPGILSMANAGPGTNGSQFFVCTAK.T + Oxidation (M)<br>K.HTGPGILSMANAGPGTNGSQFFVCTAK.T + Oxidation (M)

					K.TEWLDGKHVVFGQVVEGMDVVK.A<br>K.TEWLDGKHVVFGQVVEGMDVVK.A + Oxidation (M)<br>K.TEWLDGKHVVFGQVVEGMDVVK.A + Oxidation (M)<br>K.TEWLDGKHVVFGQVVEGMDVVK.A + Oxidation (M)<br>K.HVVFGQVVEGMDVVK.A<br>K.HVVFGQVVEGMDVVK.A + Oxidation (M)<br>R.TNKPVVIADCGQLS.-
478	stress-related protein<br>gi|116643152<br>Citrus sinensis	17593	5.67	41% / 286	K.NVELISGDGGPGSIKK.F(2+)<br>K.FNFVEGADWK.Y(2+)<br>K.IYNYTAIEGEGDANIPTIDHVSYESK.V(3+)<br>K.FYPKPGAEIKEEQVK.G(2+)
482	putativemiraculin-like protein 2<br>gi|119367468<br>Citrushybrid cultivar	23610	8.18	21%/121	R.GRNELCPLDVVQLSSDSER.G(2+)<br>R.NELCPLDVVQLSSDSER.G(2+)<br>K.IVHCPSVCESCVSLCNDVGVSNDHAR.R(3+)
488	60S ribosomal protein large subunit 9<br>gi|146454508	20965	9.70	8%/85	R.TALSHVGNLITGVTK.G(2+)

	Sonneratiaalba				
496	cysteine protease Cp<br><br>gi|151547430<br><br>Citrus sinensis	40045	6.25	20%/183	K.YNGGLDTEEAYPYTGK.D(2+)<br><br>K.CGNTPMDVNHAVVAVGYGVEDGVPYWLIK.N(3+)<br><br>K.NSWGENWGDHGYFK.I(2+)<br><br>K.NMCGIATCASYPVVA.- + Oxidation (M)(2+)
500	gibberellin 20-oxidase [Gossypiumhirsutum]<br><br>gi|222875436	43094	6.11	2%67	R.KFFEDGNSIMR.C + Oxidation (M)(2+)<br><br>K.FFEDGNSIMR.C + Oxidation (M)(2+)
510	Bisphosphate aldolase cytoplasmic isozyme-like [Citrus clementina]<br><br>gi|557532346	36597	6.96	7% 95	K.TAAGKPFVDVLK.E<br><br>K.VTPDVIAEHTVR.A
511	Fructose –bisphosphatealdolase cytoplasmic isozyme-like [Citrus clementina]<br><br>gi|557532346	36597	6.96	45% 507	K.GILAADESTGTIGK.R<br><br>K.GILAADESTGTIGKR.L<br><br>R.LSSINVENVESNR.R<br><br>K.TAAGKPFVDVLK.E<br><br>K.VDKGTVELAGTNGETTTQGLDGLAQR.C<br><br>K.GTVELAGTNGETTTQGLDGLAQR.C

					K.GTVELAGTNGETTTQGLDGLAQR.C<br>K.IGPNEPSQLAINENANGLAR.Y<br>K.ALNDHHVLLEGTLLKPNMVTPGSEAPK.V + Oxidation (M)<br>K.VTPDVIAEHTVR.A<br>K.ANSEATLGTYKGDAQLGEGAAESLHVK.D
512	Peroxidase 12-like [Citrus clementina] gi|557536631	38106	8.55	25% 343	K.GLSWTFYDQSCPK.L<br>K.DIGLAAGLIR.I<br>R.DSVALSGGPNYDLPLGR.R<br>R.ETVALSGGHTVGLAHCPAFTNR.L<br>R.QGLLTSDQDLYTDKR.T<br>K.MSQLSVLTGK.Q + Oxidation (M)
516	Cysteine proteinase RD21a-like [Vitisvinifera]gi|147790682	52761	5.10	7%/97	K.DQGSCGSCWAFSTIAAVEGINK.I(2+)<br>K.CGIAMEASYPIKK.G(2+)<br>K.CGIAMEASYPIKK.G(2+)<br>K.CGIAMEASYPIKK.G + Oxidation (M) (2+)
521	poliprotein [Mycoplasma mycoides subsp.	83310	9.13	1%/62	K.ASGSEKIDVSK.I(2+)

	mycoides SC str. PG1]gi|42561300				K.IDVSK.I(1+)
530	Adenosyl homocysteinase-like [Citrus clementina]<br><br>gi|557522891	53686	5.84	5% 69	K.GETLQEYWWCTEK.A<br><br>K.VAVVCGYGDVGK.G
533	2'-5' RNA ligase [Klebsiella pneumoniae]<br>gi|490329921	8770	10.15	18% / 66	-.MLR.A + Oxidation (M)(1+)<br><br>-.MLR.A + Oxidation (M) (1+)<br><br>-.MLRAQAAR.S(2+)<br><br>M.LRAQAAR.S(2+)<br><br>R.AQAAR.S(1+)<br><br>R.DAR.R(1+)<br><br>R.DARR.A(1+)<br><br>K.ES.- (1+)<br><br>K.ES.- (1+)
534	Calreticulin-like [Citrus clementina]<br><br>gi|557535644	41373	4.69	18% 89	K.KPDDWDDEEDGEWTAPTIPNPEYK.G<br><br>K.APMIDNPDFKDDPDLYVYPNLK.Y + Oxidation (M)<br><br>K.SGTMFDNVLVSDDPEYANK.L + Oxidation (M)
542	Rubisco gi|134101	52461	4.77	6%/98	K.TNDSAGDGTTTASVLAR.E(2+)

	Ricinus communis				R.ELAETDSVYDSEK.L(2+)
